# Platelet-Rich Fibrin and Its Emerging Therapeutic Benefits for Musculoskeletal Injury Treatment

**DOI:** 10.3390/medicina55050141

**Published:** 2019-05-15

**Authors:** Alexandru Florian Grecu, Lucien Reclaru, Lavinia Cosmina Ardelean, Oliviu Nica, Eduard Mihai Ciucă, Marius Eugen Ciurea

**Affiliations:** 1 University of Medicine and Pharmacy Craiova, 200349 Craiova, Romania; alexandrugrecu@yahoo.com; 2 Varinor Matériaux SA, CH 2800 Delémont, Switzerland; lucien.reclaru@varinor.ch; 3 Department of Technology of Materials and 9 Devices in Dental Medicine, “Victor Babes” University of Medicine and Pharmacy from Timisoara, 300041 Timisoara, Romania; lavinia_ardelean@umft.ro; 4 Department of Oro-Maxilo-Facial Surgery, University of Medicine and Pharmacy Craiova, 200349 Craiova, Romania; 5 Department of Plastic Surgery, University of Medicine and Pharmacy of Craiova, 200349 Craiova, Romania; meciurea@gmail.com

**Keywords:** platelet-rich fibrin, musculoskeletal system, biological therapy, bone regeneration, platelet activation

## Abstract

New therapies that accelerate musculoskeletal tissue recovery are highly desirable. Platelet-rich fibrin (PRF) is a leukocyte- and platelet-rich fibrin biomaterial that acts as a binding site for both platelets and growth factors. Through increasing the local concentration of growth factors at specific tissues, PRF promotes tissue regeneration. PRF has been frequently used in combination with bone graft materials to reduce healing times and promote bone regeneration during maxillofacial surgery. However, its benefits during muscle repair and recovery are less well-documented. Here, we perform a narrative review on PRF therapies and muscle injuries to ascertain its beneficial effects. We reviewed the factors that contribute to the biological activity of PRF and the published pre-clinical and clinical evidence to support its emerging use in musculoskeletal therapy. We include in vitro studies, in vivo animal studies and clinical articles highlighting both the success and failures of PRF treatment. PRF can promote the healing process when used in a range of orthopaedic and sports-related injuries. These include cartilage repair, rotator cuff surgery and anterior cruciate ligament surgery. However, conflicting data for these benefits have been reported, most likely due to inconsistencies in both PRF preparation protocols and dosing regimens. Despite this, the literature generally supports the use of PRF as a beneficial adjuvant for a range of chronic muscle, tendon, bone or other soft tissue injuries. Further clinical trials to confirm these benefits require consistency in PRF preparation and the classification of a successful clinical outcome to fully harness its potential.

## 1. Introduction

Autologous platelet concentrates are simple and cost-effective methods that allow high local concentrations of growth factors at a target tissue [1]. Platelet-rich fibrin (PRF) is a second-generation platelet concentrate developed by Choukroun et al. because of legal restrictions on blood handling. PRF contains platelets, leukocytes, cytokines and adhesive proteins including fibrinogen, fibronectin, vitronectin, and thrombospondin-1 [2,3]. The presence of white blood cells (WBCs), that secrete a large quantity of growth factors, is a key feature of PRF which is named for this reason leukocyte-PRF (L-PRF). The polymerization of the fibrin produces a 3-dimensional cross-linked fibrin matrix [4,5,6,7,8]. This serves as a binding site for both platelets and growth factors [9,10]. By increasing the local concentration of growth factors at a specific tissue location, PRF promotes tissue regeneration by closely mimicking the wound repair process over a prolonged period of time [2,3,11,12,13,14]. The ultimate result is a healing matrix that possesses unique mechanical properties, making it distinct from platelet-rich plasma (PRP) and other platelet concentrates [15,16,17]. Herein we discuss the promising clinical benefits of PRF with a focus on its ability to promote wound and muscle healing.

### 1.1. Muscle Injury and Repair 

Muscle repair is an evolutionarily conserved multicellular process requiring the synchronized activity of infiltrating macrophages, resident myogenic and non-myogenic stem cells, and connective tissue fibroblasts [18]. Despite the effectiveness of this intrinsic repair mechanism, severe injuries that result in a significant loss of muscle tissue and/or a loss of tissue function overwhelm the repair process and require therapeutic intervention. Musculoskeletal disorders are classed as any injury, damage or disorder of the joints or tissues in the upper/lower limbs or the back [19]. The World Health Organization (WHO) reports that musculoskeletal injuries affect the lives of hundreds of millions globally, and are the major worldwide cause of long-term pain, trauma and physical disability [20,21,22]. Soft tissue injuries comprise 40–50% of musculoskeletal problems and are typically the result of cartilage, ligament and tendon damage. Severe impact injuries, arthritis and osteoporosis are examples of chronic and degenerative musculoskeletal conditions that pose severe challenges for clinicians [23]. Chronic injuries, including those resulting from sports- and overuse-related injuries, often require surgery leading to prolonged recovery times. In addition, a successful outcome and complete recovery following surgery are often not guaranteed and current adjuvant therapies that aid the healing process remain limited [19,20].

Hope has been offered through the discovery of orthobiologics. These refer to the use of biological substances that improve the healing of injured muscles, tendons and ligaments [24]. Orthobiologics are derived from substances naturally found in the body. They include bone grafts, autologous blood, PRP, autologous conditioned serum and stem cells [24,25,26,27,28]. The benefits of using orthobiologics are the reduced need for surgery when treating musculoskeletal injuries and to augment the effectiveness of existing surgical techniques [28]. Autologous blood, PRP and conditioned serum work by delivering growth factors to the injured area, stimulating the repair process [24,25]. Autologous blood products have surpassed the use of recombinant growth factors for this purpose due to their lower cost, longer life span, and more efficient delivery to the target tissue [24].

### 1.2. Orthobiologics 

Autologous blood injections were first used to deliver blood-derived growth factors to injured tissue. This involved drawing blood from a patient and injecting it into the desired area. This procedure delivers growth factors contained in platelets. This method has been questioned, with both positive [29,30,31] and negative results reported [32]. The knowledge of growth factors being primarily contained in the alpha granules of platelets led researchers to focus on more effective growth factor delivery methods [33]. These have evolved from the use of autologous whole blood to the use of concentrated platelets, otherwise known as PRP. PRP is defined as a plasma fraction of autologous blood with a platelet concentration above baseline levels [34]. Despite some clear benefits, solid evidence in favor of its clinical application to trauma and orthopaedic surgery is still lacking [25]. PRP has been commercialized, yet it contains products that are unnatural and, in some instances, can inhibit wound healing. By removing these anti-coagulants and modifying the blood collection protocols, PRF was introduced some years later, with the exciting potential to markedly improve surgical performance.

## 2. Methods—Literature Review

We searched several databases including Pubmed and MEDLINE as well as reference lists of relevant articles to compile a consensus for the role of PRF and its therapeutic effectiveness. A Pubmed search of “platelet-rich fibrin” yielded 783 peer-reviewed articles that were filtered for relevance. The criteria for inclusion were (1) in vitro studies that report the effects of PRF on cell lines or cultures of primary cell lines in vitro, (2) in vivo animal studies evaluating the effect of PRF on muscle repair, (3) Clinical articles highlighting both successes and failures of PRF treatments. Through MEDLINE, the number of PRF clinical trails to date are recorded as 114 (clinicaltrails.gov). The selected studies were reviewed and their reference lists checked for all other eligible articles. Keywords used in this review include PRF and: maxillofacial surgery, muscle, cartilage, rotator cuff surgery, hamstring injury.

### 2.1. Platelet Rich Fibrin

PRF represents a significant advance in the evolution of platelet concentrates. In its simplest form, PRF is a fibrin membrane with trapped platelets ± leukocytes [22,25,33,35,36,37,38,39,40,41,42]. These solid membranes display excellent handling characteristics, and can be firmly sutured in an anatomically desired location during open surgery [43,44,45,46,47,48,49,50,51,52,53]. PRF has generated much excitement through its potential benefits to wound healing and tissue injury, which will be discussed herein. Ease of preparation, low cost, minimal risks for the patients, and possible outpatient use make PRF an optimal scaffold for tissue healing processes [54].

### 2.2. Preparation of PRF 

A major advantage of PRF is the simplicity of preparation. PRF is drawn from the blood of a patient using a sterile 10 mL vacutainer either before, during or after surgery [2,3,11,12,13]. Blood is collected into a vacuum-sealed tube without anticoagulants and centrifuged for 12 minutes at 2700 rpm to separate red blood cells and plasma from platelets [55,56]. The fibrin clot containing the platelets is located in the center of the tube, between the lower layer of RBCs and the plasma acellular on the top. The PRF produced is free of anticoagulants and/or other artificial biochemical modifications [2,3,11,12,13,57]. Changing the PRF centrifugation protocol can influence both the structure and density of PRF clots [58,59]. The clinical efficacy of centrifugation protocols still requires validation, as open access methods have led to many variations of the original PRF protocol that have confused the literature [54,60]. This presents a problem as the material produced from each protocol differs from that of the original PRF [54,55,61,62]. These include differences in the size and weight of the clots/membranes leading to varying biological signatures and clinical outcomes [54]. To date, the differences between the methods of PRF production and the biological characteristics of final PRF product have not been clearly characterized. Standardization of centrifugation protocols to control growth factor content and fibrin architecture are now required, and the effects of modifications of the original production protocol on PRF activity require clarification [54].

### 2.3. Handling and Application of PRF

PRF membranes can be easily departed over a surgical or augmented site. Due to its elastic consistency, a clinician can puncture a hole in the membrane which can be draped over a healing area. PRF is blood-derived living tissue, so it must be handled accordingly. As PRF lacks anticoagulants, blood samples will coagulate upon contact with the tube glass. Quick handling ensures a clinically usable PRF matrix. If the duration between blood collection and centrifugation is too long, PRF samples become inconsistent. By driving out the fluids trapped in the fibrin matrix, very resistant autologous fibrin membranes can be obtained. Advanced platelet-rich fibrin (A-PRF) defines a new set of centrifugation speeds/times that prevent cell loss within the PRF matrix [63]. A-PRF is richer in WBCs compared to PRF and displays improved collagen matrix synthesis and enhanced recruitment of progenitor cells [62,64]. Injectable platelet-rich fibrin (i-PRF) was developed to act as a regenerative agent that could be delivered in liquid formulation [65,66]. To achieve this, blood is drawn rapidly in a specific centrifugation tube at a low speed (approximately 700 rpm for 3 minutes). I-PRF can be mixed with bone grafts to form a stable fibrin-graft that also improves wound healing [64,65].

### 2.4. PRF Growth Factors

Kobayashi et al. performed a comparison of growth factor release for PRP, PRF, and A-PRF over time [67]. The various platelet concentrates were shown to have differing growth factor release kinetics. PRP released significantly higher levels of growth factors at earlier time points, whilst PRF displayed continual and steady release over a 10-day period. A-PRF released significantly higher quantities of growth factors when compared to traditional PRF [66]. From the same samples of peripheral blood, Qiao et al. compared the levels of five important growth factors in activated PRP and PRF by ELISA (enzyme-linked immunosorbent assay) [68]. The results revealed that the levels of basic fibroblast growth factor (bFGF) in PRF were significantly higher than that in activated PRP. For the other growth factors tested, namely platelet-derived growth factor-BB (PDGF-BB), transforming growth factor β-1 (TGF-β1), insulin-like growth factor-1 (IGF-1), and vascular endothelial growth factor (VEGF), no significant differences between the two platelet concentrates were observed [67]. PRP is thus recommended for the fast delivery of growth factors whist PRF and A-PRF are better suited for long-term growth factor release.

### 2.5. Uses of PRF 

#### 2.5.1. Oral and Maxillofacial Surgery

Before discussing muscle injury, we first discuss other widely accepted benefits of PRF, particularly during oral and maxillofacial surgery. PRF membranes are frequently used in combination with bone graft materials to reduce healing times and promote bone regeneration. Choukroun et al. showed that PRF with freeze-dried bone allograft increased bone formation during sinus lift procedures [2]. These findings were confirmed and more efficient methods to alleviate the postoperative complications of this procedure using PRF evolved [69,70]. PRF was also used with success during the treatment of Intrabony periodontal defects [71,72,73]. Greater attachment levels and bone-fill were notable findings. Similar improvements in patient outcomes were observed for mandibular Grade II furcation defects [74] and gingival recession defects [75,76,77,78,79,80,81]. Its ability to relieve pain and post-operative swelling during tooth extractions and in the treatment of tooth lesions, are also widely documented [82,83,84,85,86,87,88,89,90,91,92]. PRF was also used in the treatment of medication-related osteonecrosis of the jaw (MRONJ), with promising results for the healing and quality of life improvement of patients with MRONJ [93].

#### 2.5.2. Orthopaedics and Sports Injuries

Although skeletal muscle can respond to a range of environmental changes, the appropriate treatment for chronic muscle injuries remains a clinical challenge. Healing with conventional therapies is frequently insufficient, leading to substantial interest in the potential of platelet concentrates to aid muscle recovery. The process of muscle healing and the contribution of growth factors to the healing process is an intensively studied field and is reviewed in [21,22]. By concentrating these growth factors, PRF can promote the cellular events required for muscle repair and regeneration [94]. Some of the first studies to reveal this were performed by Wright-Carpenter and co-workers who assessed the effects of PRF on muscle strain injuries in humans. Subjects were treated with PRF two days after injury and every two days thereafter [21]. The study revealed that the recovery time of the PRF-treated subjects was reduced to 16.6 days compared to 22.3 days in subjects treated with anti-inflammatory treatments. This supported the use of PRF during muscle therapy, validating previous observations from animal models and in vitro studies.

#### 2.5.3. Cartilage Injuries

In the past decade, PRF has emerged as a promising non-operative procedure for the treatment of cartilage injuries. Chien and colleagues were amongst the first to assess the incorporation of PRF into biodegradable fibrin (FB) scaffolds as a regeneration matrix for promoting chondrocyte proliferation and re-differentiation [95]. Chondrocytes are the only cells found in healthy cartilage and are responsible for the production of the cartilaginous matrix. It was concluded that FB scaffolds promoted proliferation and re-differentiation that was enhanced by PRF, offering promise for cartilage tissue engineering. PRF also aids the healing of knee cartilage defects in animal models [96,97]. Defects in the left hind limbs of dogs receiving PRF implantation were found to display improved articular cartilage repair [16,98,99]. Studies in rabbits also demonstrated the ability of PRF to enhance the viability of diced cartilage grafts, promoting its use as an appropriate biological wrapping material to aid cartilage grafting [100,101]. More recent studies reveal that cartilage regeneration improves more significantly using i-PRF as opposed to autologous PRF [58]. Complicated procedures requiring in vitro chondrocyte expansion prior to cartilage repair were also shown to be avoidable through the development of a culture-free, single-stage approach that combines PRF with autologous cartilage grafts [96].

#### 2.5.4. Rotator Cuff Repair 

Rotator cuff tears are a common cause of shoulder pain and disability and can be caused by traumatic and degenerative elements. This frequently makes surgical repair challenging as the original tendon-bone insertion is difficult to recreate. Accordingly, non-healing rates are reported to be as high as 94% [36]. Zumstein and colleagues showed that l-PRF delivered in a standard/gelatinous-type matrix could locally deliver healing growth factors for up to 28 days, aiding rotator cuff repair [36]. In later pilot trials, when l-PRF was added in between tendon and bone during arthroscopic rotator cuff repair, the vascularization index improved in contralateral healthy shoulders at 6 and 12 weeks, with no post-operative complications reported [102]. Thus, arthroscopic rotator cuff repair using l-PRF appeared technically feasible, supporting earlier in vitro studies that demonstrated its ability to enhance tenocyte proliferation and promote extracellular matrix synthesis. Despite this progress, later studies questioned the clinical benefits of PRF for more severe muscle rotator cuff injuries. When PRF was applied to massive rotator cuff tears, two independent studies revealed no changes to anatomic healing rates, mean postoperative defect sizes and tendon quality [103,104]. Hasan and colleagues also showed that PRF induces a disordered healing response characterized by fibro vascular scar tissue in rat models of severe rotator cuff repair [105]. Further studies are therefore required to clarify the benefits of PRF during this procedure.

#### 2.5.5. Other Sports-Related Injuries

The success of PRF augmentation was noted by Alviti and colleagues who assessed its effects on ankle stiffness and the mechanical work of the ankle in patients who had undergone Achilles tendon surgery [106]. The findings suggested that treatment with a suture and PRF resulted in a significant functional improvement in terms of the efficiency of motion, improving patient recovery. Similar benefits were reported for operations involving the replacement of torn anterior cruciate ligaments (ACL) [107]. Most of the patients had normal knee scores and returned to normal levels of sporting activity when PRF was used. In other studies, PRF augmentation did not appear to have an effect on gluteus medius tendon repair in terms of pain or clinical evidence, but was shown to improve hip-specific physical functioning following surgical repair [108]. The potential benefits of PRF to elite-level athletes were also highlighted in randomized control trials that assessed its effects on ankle sprains. Ultrasound-guided injections of PRF into the injured antero-inferior, tibio-fibular ligaments, led to benefits that included a shorter recovery, re-stabilization of the syndesmosis joint and reduced long-term pain [109]. Beneficial effects of PRF have also been reported in cases of patellar tendinopathy [110], partial ulnar collateral ligament tears [111], elbow ulnar collateral ligament insufficiency in high-level throwers [112], and in the treatment of meniscal lesions [113]. The promise of PRF is not limited to muscle injuries, and recent studies reveal its ability to promote bone healing and regeneration when applied with several grafting materials in animal and human calvarial defects [114,115,116]. Thus, iPRF offers hope to aid both bone and muscle regeneration following serious trauma-induced injuries.

## 3. Conclusions

The articles included and presented in this review were chosen due to the clarity of proof, concise and clear results. The articles that were disregarded either presented inadequate information about the topic or they were considered as lacking clarity.

Despite the vast majority of articles proving the benefits of PRF to promote tissue regeneration and speed up recovery, there is still some controversy in the literature regarding this. Recent controlled clinical trial data provides hope that PRF benefits cartilage injury, whilst its use is supported for partial ulnar collateral ligament tears, elbow ulnar collateral ligament insufficiency and in the treatment of meniscal lesions. The literature for other muscle interventions has been conflicting as well (exemplified by rotator cuff injuries), most likely due to the fact that not all PRFs are the same and those with higher leukocyte levels may be detrimental to tendon healing. To date, the clinical data do not conclusively prove the benefits of PRF treatment to aid muscle injury.

Consistent dosing and reproducible measures of success outcomes are now required to definitively prove effectiveness. Whether our scientific ability to fully harness its potential is still lacking remains an important question to consider during future investigations.
